# Underserved Patient Populations With Metastatic Breast Cancer: A Review of Progress and Remaining Challenges

**DOI:** 10.1155/tbj/2461234

**Published:** 2025-06-30

**Authors:** Fatima Cardoso, Rachel Wuerstlein, Tomoyuki Aruga, Renate Haidinger, Matteo Lambertini, Christine Benjamin, Elisenda Llabrés Valentí, Carmen Criscitiello, Matti Aapro, Generosa Grana, Sharon S. Gentry, Eduard Vrdoljak

**Affiliations:** ^1^Advanced Breast Cancer Global Alliance, Lisbon, Portugal; ^2^Department of Obstetrics and Gynecology, Ludwig Maximilian University, Munich, Germany; ^3^Department of Surgery, Institute of Science Tokyo, Tokyo, Japan; ^4^German Breast Cancer Association, Hohenbrunn, Germany; ^5^Department of Internal Medicine and Medical Specialties, University of Genoa, Genoa, Italy; ^6^Department of Medical Oncology, Ospedale Policlinico San Martino, Genoa, Italy; ^7^SHARE Cancer Support, New York, New York, USA; ^8^Department of Medical Oncology, Insular University Hospital of Gran Canaria, Las Palmas de Gran Canaria, Spain; ^9^Department of Oncology and Hematology, University of Milan, Milan, Italy; ^10^Division of New Drugs and Early Drug Development, European Institute of Oncology, Milan, Italy; ^11^Department of Medical Oncology, Genolier Cancer Center, Genolier, Switzerland; ^12^MD Anderson Cancer Center at Cooper, Cooper University Health Care, Camden, New Jersey, USA; ^13^Academy of Oncology Nurse & Patient Navigators, Cranbury, New Jersey, USA; ^14^Department of Oncology, University of Split, Split, Croatia

**Keywords:** advanced breast cancer, health disparities, health inequity, metastatic breast cancer, unmet needs

## Abstract

Breast cancer presents a significant risk to public health and is the primary cause of cancer-related death in women. Awareness of metastatic breast cancer (mBC) continues to increase, and advances have been made; however, challenges remain for many patient populations that do not receive equal opportunities along the treatment pathway. The Underserved Patient Population (UPP) Coalition Task Force, a group of international experts in mBC, held meetings between 2022 and 2023 to prioritise the needs of UPPs and propose solutions. The key unmet needs identified included the following: delayed diagnosis of mBC due to difficulties in the presentation of patients to the healthcare system and a lack of primary care physician and non–breast cancer specialist understanding of the signs and symptoms of mBC; difficulty navigating the mBC patient pathway due to suboptimal use of multidisciplinary care and limited communication between HCPs; unequal access to the most appropriate mBC treatment options and supportive therapy due to the unconscious bias of HCPs, and direct and indirect financial toxicity for patients; and negative impact on QoL resulting from the limited uptake of shared decision-making, low prioritisation of patient preferences and a lack of personalised care. This paper aims to shine light on initiatives supporting underserved patients with mBC, illustrate the remaining gaps in care and call upon the global community to change how care is delivered to UPPs.

**Summary**

• Oncologists were surveyed to quantify disparities in metastatic breast cancer care
• Disparities in metastatic breast cancer care, diagnosis and treatment remain
• Multidisciplinary experts proposed solutions to improve metastatic breast cancer care
• New tools were developed to reduce disparities in metastatic breast cancer care

## 1. Introduction

Breast cancer is the most frequently diagnosed cancer and the leading cause of cancer-related death in women, with approximately 2.3 million new cases and 685,000 deaths reported globally in 2020 [1]. Outcomes for patients with breast cancer have improved in recent decades, with a 5-year relative survival rate of over 90% for Stages I and II; however, patients with metastatic breast cancer (mBC) continue to have poor outcomes and an associated 5-year survival rate of 27%–34% [2].

Between 20% and 30% of early breast cancer cases will progress to mBC [3]. Furthermore, an increasing number of patients with no history of early breast cancer are diagnosed with *de novo* mBC [4]. mBC impacts the lives of many patients and their caregivers and continues to represent a significant global health and social concern.

In recent years, great advances have been made in mBC due to the development of innovative treatments for all cancer subtypes, including several anti-HER2 agents for HER2+ mBC, cyclin-dependent kinase (CDK) 4/6 inhibitors, phosphatidylinositol 3-kinase (PI3K) inhibitors, selective oestrogen degraders and other endocrine-based therapies for HR+ HER2− mBC, immune checkpoint inhibitors and antibody–drug conjugates for triple-negative mBC and polyadenosine diphosphate ribose polymerase (PARP) inhibitors for BRCA-associated mBC. These treatments provide targeted and more individualised options for patients, which can improve survival outcomes [5]. However, mBC remains an incurable disease in most cases [6, 7]. Moreover, barriers in access to diagnostics, treatments and healthcare services mean that innovative therapies do not reach all patients, resulting in disparities in mBC care [8–11]. A pan-European multidisciplinary expert meeting was held in 2019 to identify mBC patient populations who were less likely to receive quality healthcare due to individual or system-related factors. These groups of patients are referred to as underserved patient populations (UPPs).

The outcomes of the multidisciplinary meeting, supplemented by a literature review and mixed-method survey of meeting attendees, led to the identification of five key characteristics of UPPs with mBC: older patients; patients from ethnic, religious, indigenous or native populations and/or other minorities; low-income patients; patients with low health knowledge; and patients living a long distance from a specialist centre [8]. Experts also discussed and proposed solutions to the challenges faced by UPPs with mBC, publishing the conclusions in 2021 [8].

Local and regional efforts to overcome these challenges have been driven by patient advocacy groups, healthcare professionals (HCPs) and the pharmaceutical industry. In 2020, the Advanced Breast Cancer (ABC) Global Alliance and Pfizer developed the Hard-to-Reach ABC/mBC Communities Toolkit, a repository of best practice initiatives for UPPs with mBC, selected examples of which are discussed throughout this publication [12, 13]. Despite these efforts, a quantitative analysis conducted by Ipsos in Europe and Japan found that disparities in mBC detection, diagnosis and treatment still exist [14], and recent studies in the United States, Brazil and Ethiopia highlighted disparities in these countries, indicating the significant unmet needs that remain for UPPs with mBC worldwide [9, 15–18].

This article aims to define key UPPs with mBC and their prevalence, describe the key unmet needs and challenges identified by the UPP Coalition Task Force and highlight best practice initiatives to help address inequities in care for these groups.

## 2. Methods

### 2.1. Quantitative Research and Analysis

In 2020, Ipsos conducted the Pfizer-funded HR+ HER2− mBC UPP sizing analysis to understand the prevalence of UPP characteristics in patients with HR+ HER2− mBC [19] and disparities in access to treatment for these patients [20]. The gaps and barriers identified in the HR+ HER2− subgroup were deemed applicable to all patients with mBC, as this is the most common subtype, for which various new treatments have recently been developed [5, 21, 22]. The survey was completed by gynaecological, medical and haematological oncologists, and breast surgeons, from the EU4 countries, the UK and Japan (Table 1). Respondents were compensated for completing the survey. The data on treatment differences were updated in 2022.

In 2022, Ipsos conducted an additional Pfizer-funded quantitative survey: mBC analysis, to build on insights from the previous research and obtain additional information about UPPs with mBC and disparities in breast cancer detection and mBC care (Table 1) [14].

The findings of these analyses were used to stimulate discussion during the UPP Coalition Task Force meetings.

### 2.2. UPP Coalition Task Force

The UPP Coalition Task Force was established in 2022. Members included a diverse range of roles (Table A1) and were invited based on their expert, multifaceted knowledge of mBC and prior engagement in health equity initiatives. Several of the Task Force members previously attended the original multistakeholder group meeting in 2019.

Insights informing the development of this publication were generated from four Task Force meetings: three with experts from Europe, Japan and the United States, respectively, and a global meeting including Task Force members from each of these countries in addition to new members including a specialist social worker and policy experts, to diversify perspectives.

Each meeting followed a similar format: a quantitative survey was disseminated in advance, and the responses were presented alongside recent data relevant to UPPs with mBC and followed by a facilitated discussion on key topics identified. Initial meetings focussed on determining unmet needs and challenges for UPPs, with later sessions used to ideate, prioritise and discuss the implementation of solutions to overcome these challenges. Each meeting was a progression from the last, resulting in an evolution of findings and priorities for the Task Force over time.

### 2.3. Supplementary Literature Search

The key unmet need themes identified were validated by a literature search, including the analysis of several sources relevant to global unmet needs for patients with mBC: review articles, clinical trial results, country policies and guidelines, patient advocacy group websites, white papers and consensus statements—published between 2018 and October 2023. Select earlier pieces of the literature (from 2011 onwards) were used to validate perspectives and insights from Task Force members.

## 3. Results and Discussion

### 3.1. Findings of the Ipsos Sizing Analysis and mBC Analysis

**Figure 1:**
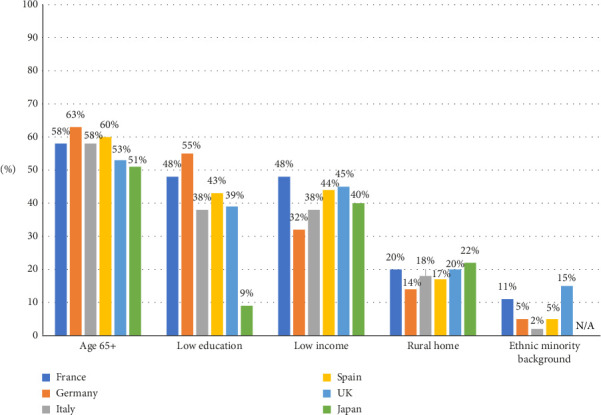
Prevalence of key UPP characteristics amongst patients with HR+ HER2− mBC in six countries [19, 20]. Abbreviations: N/A: not applicable and UK: United Kingdom. Proportion of patients with HR+ HER2− mBC with UPP characteristics based on Pfizer-funded survey results collected from oncology physicians by Ipsos in 2020. Ethnic minority background was not studied in Japan. This figure was produced by the authors for the purpose of this publication.

The HR+ HER2− mBC UPP sizing analysis identified old age as the most prevalent UPP characteristic among patients with HR+ HER2− mBC in all studied countries (51%–63%). In all countries other than Japan, a sizeable proportion (32%–55%) of patients with HR+ HER2− mBC were estimated to have low income or low education level. Ethnic minorities were reported as the smallest UPP group, with less than 15% in all studied countries (Figure 1) [19, 20].

The impact of age and ethnicity on the treatment of patients was determined via an analysis of Ipsos Oncology Monitor data and physician survey outputs. In all studied countries, patients above 75 years were less likely to receive CDK 4/6 inhibitors—the standard-of-care first-line treatment option recommended by all international and national guidelines for patients with HR+ HER2− mBC [23–26]—compared to younger patients [19, 23–27]. Racial disparities were also observed, with non-Caucasian patients less likely than their Caucasian counterparts to receive CDK 4/6 inhibitors in France, Germany, Spain and the UK [19, 27]. Further analysis in 2022 found that patients with HR+ HER2− mBC above the age of 50 years in the EU5 and Japan were less likely to receive chemotherapy than younger patients [19, 28].

In the mBC analysis study, ‘fear of the outcome or denial that there may be a problem' was identified as a key barrier to a timely breast cancer diagnosis. Low health knowledge and older age were also found to considerably impact diagnosis, especially in Germany and the UK (Table A2). Meanwhile, HCPs from Japan highlighted that divorced and widowed women with mBC are at high risk of being underserved [14]. Existing comorbidities, a lack of caregiver or support system, low health knowledge, age and low income were identified as factors that may impact HCPs' decision to prescribe CDK 4/6 inhibitors for patients with HR+ HER2− mBC (Table A3) [14].

### 3.2. Key UPP Characteristics Identified by the UPP Coalition Task Force

Further to those identified in 2019, the Task Force suggested additional UPP characteristics based on personal experience and the findings of the Ipsos research analyses (Table 2).

### 3.3. Key Unmet Needs for UPPs With mBC Identified by the UPP Coalition Task Force

**Figure 2:**
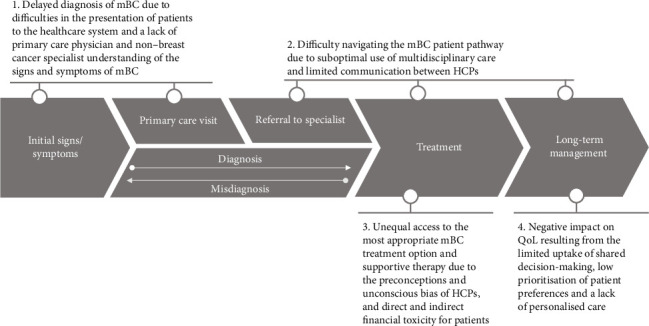
Key unmet need areas for UPPs with mBC prioritised by the UPP Coalition Task Force between 2022 and 2023. Abbreviations: HCP: healthcare professional, mBC: metastatic breast cancer, QoL: quality of life and UPP: underserved patient population. This figure was produced by the authors for the purpose of this publication.

The UPP Coalition Task Force identified and prioritised four key unmet need areas for UPPs with mBC (Figure 2) and ideated potential solutions to overcome these.

#### 3.3.1. Delayed Diagnosis of mBC due to Difficulties in the Presentation of Patients to the Healthcare System and a Lack of Primary Care Physician and Non–Breast Cancer Specialist Understanding of the Signs and Symptoms of mBC

Low awareness and understanding of mBC can delay patient presentation to health systems [30]. Symptom recognition can be challenging for people with low health knowledge, who may not be aware that certain common symptoms, for example, fatigue, nausea or pain, can be indicative of mBC [31, 32]. Even when symptoms are recognised, some patients may delay seeking help due to fear of an mBC diagnosis and mortality, or concerns about mBC treatment costs [14, 33]. The logistical burden of travelling can be especially challenging for patients who live far from a healthcare centre, further limiting patient presentation [34]. In certain communities or cultures, stigma associated with breast cancer can result in the isolation or blaming of patients, which may also discourage presentation to healthcare systems [35, 36].

Delays to diagnosis may also occur due to a lack of awareness of mBC signs and symptoms among primary care physicians and non–breast cancer specialists, such as gynaecologists or dermatologists, who may only receive limited oncology training [37]. The overlap of mBC symptoms with common ailments, such as headaches and bone pain, further adds to this challenge [38].

##### 3.3.1.1. Existing Initiatives That Aim to Overcome This Need and Additional Solutions Proposed and Created by the UPP Coalition Task Force

Initiatives have been developed to improve public and HCP awareness and understanding of mBC. Select examples are highlighted in Table A4, many of which are featured in the ABC Global Alliance and Pfizer's Hard-to-Reach ABC/mBC Communities Toolkit [13]. However, very few initiatives exist to educate primary care physicians and non–breast cancer specialists on mBC and its signs and symptoms. In 2022-23, the expert group co-created two innovative resources that were then developed by Pfizer to help overcome this need. These tools are endorsed by the ABC Global Alliance and are available on their website:

• Educational Toolkit for Primary Care Physicians and Non–Breast Cancer Specialists
• The Patient Barrier Assessment Tool

###### 3.3.1.1.1. Educational Toolkit for Primary Care Physicians and Non–Breast Cancer Specialists

**Figure 3:**
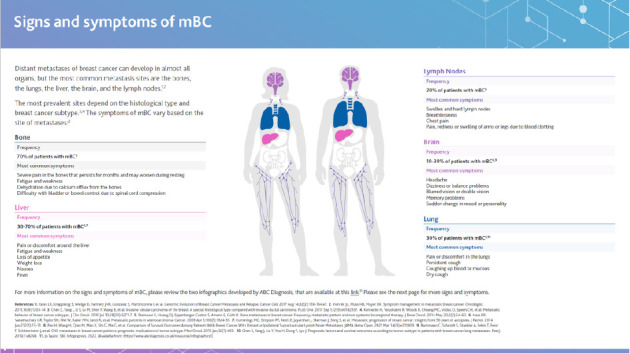
Example page of the educational toolkit for primary care physicians and non–breast cancer specialists: signs and symptoms of mBC. Reproduction of the educational toolkit for primary care physicians and non–breast cancer specialists, with permission from the UPP Coalition Task Force, ABC Global Alliance and Pfizer.

This toolkit contains guidance for recognising the signs and symptoms of mBC and highlights the UPP characteristics identified during the Task Force meetings (Figure 3). It also includes suggestions for improving communication with UPPs and providing them with support to navigate their diagnosis and treatment.

###### 3.3.1.1.2. Patient Barrier Assessment Tool

**Figure 4:**
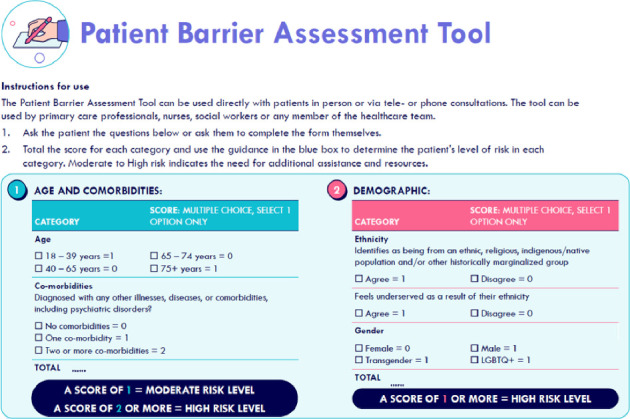
Example page of the Patient Barrier Assessment Tool: identifying patients at risk due to age, comorbidities or demographics. Reproduction of the Patient Barrier Assessment Tool, with permission from the UPP Coalition Task Force, ABC Global Alliance and Pfizer.

The Patient Barrier Assessment Tool is intended as a quick triage resource for HCPs to identify patients who are at risk of being underserved due to potential unconscious biases in clinical decision-making. It contains a checklist of questions to determine an individual's risk of facing disparities, including their age, demographic, language barriers, health knowledge, distance from health centres, financial barriers and difficulties with using technology. Each question has an associated scorecard, with guidance to determine whether a patient has high risk of being underserved in each category (Figure 4). The tool supports timely detection of underserved patients and the provision of additional support for those considered high risk.

###### 3.3.1.1.3. Other Proposed Solutions

Although mBC awareness campaigns exist, the Task Force identified the need to employ information in multimedia formats to reach different UPP groups. While social media is effective for some, newspaper advertisements, television campaigns or books may be optimal for elderly patients and local events for rural or ethnic communities [39]. Furthermore, there is a need for sensitive patient education on the potential for recurrence following the treatment of primary breast cancer, to facilitate timely mBC diagnosis. Campaigns should include a diverse set of patients, to ensure relatability and empower UPPs to present to healthcare systems sooner. As many symptoms of mBC are similar to other common illnesses [38], campaigns should be designed to minimise unnecessary fear that could overwhelm healthcare systems.

Initiatives targeting broader audiences may also be beneficial. For example, educating men to better support relatives or friends living with mBC to identify potential symptoms of the disease [40, 41]. Furthermore, the Task Force proposed the creation of a children's book or comic featuring a character living with mBC, inspired by the Breast Cancer Patient Education Novelas featured in the Hard-to-Reach ABC/mBC Communities Toolkit (Table A4), to both increase public awareness and support patients when explaining their diagnosis to family.

Finally, to assess the impact of such initiatives on delayed diagnosis of mBC, the Task Force proposed country-level monitoring of the time between initial patient presentation and confirmed diagnosis, with targets developed by Centres of Excellence.

#### 3.3.2. Difficulty Navigating the mBC Patient Pathway Due to Suboptimal Use of Multidisciplinary Care and Limited Communication Between HCPs

The mBC treatment pathway is complex, with patients often needing to attend multiple appointments with different HCPs to address varying aspects of their care [42]. This may be particularly challenging for patients with low health knowledge, who often have limited access to easy-to-understand information and patient navigation services. UPPs may also have lower awareness of and access to supportive and palliative care, including mental health support.

In most countries, HCPs have limited consultation time with patients, which can significantly impact the quality of their communication [42]. Specialist oncology nurses help to overcome this by providing additional information and supportive care to patients [43], but there is a need for more in the healthcare system globally [44–46]. A lack of time can also hinder communication between HCPs [47]. This can result in inadequate multidisciplinary care, which can be especially impactful for mBC patients with comorbidities [24]. Moreover, primary care physicians may lack clarity on the most relevant and important information to share with oncologists. The Task Force highlighted that limited communication between oncology specialists and primary care providers can result in early breast cancer survivors becoming lost in follow-up, or older patients who stop having mammograms becoming lost from the healthcare system, resulting in delayed mBC diagnosis and care for these patients.

##### 3.3.2.1. Existing Initiatives That Aim to Overcome This Need and Solutions Proposed by the UPP Coalition Task Force

Initiatives and services [12, 48–52] exist to help patients and HCPs navigate the mBC treatment pathway, including many that are featured in the Hard-to-Reach ABC/mBC Communities Toolkit [13], a selection of which are listed in Table A5. The UPP Coalition Task Force concluded that investment in the training of community navigators could enhance support for mBC patients with transportation needs, direction to useful resources and facilitating connections to advocacy or peer support groups. Patients living long distances from treatment centres may be more easily reached by navigators via telephone consultations. Finally, the Task Force noted that education and navigation support for early breast cancer survivors on the potential reoccurrence of the disease could improve the attendance of these patients at follow-up appointments.

The *Alerta Rosa* Navigation programme in Mexico represents a successful example of navigation support for underserved communities [53]. Through this programme, people with concerns about breast cancer can contact a qualified navigator, who offer guidance and support to schedule timely diagnostic and follow-up appointments [53]. Navigators confirm patient attendance at appointments and support with rescheduling missed consultations. They can also connect patients to additional services, such as social workers [53]. This programme has enhanced health system efficiencies, improving the timely diagnosis and treatment of people with breast cancer in Mexico. Learnings from this initiative should be leveraged for similar programmes in mBC [53]. Very few initiatives exist to improve communication between HCPs and enhance multidisciplinary team (MDT) working. To enhance patient and HCP understanding of multidisciplinary mBC care, the Task Force proposed the development of an educational resource outlining the different roles of an mBC MDT and indicating relevant additional services based on individual patient needs.

#### 3.3.3. Unequal Access to the Most Appropriate mBC Treatment Options and Supportive Therapy Due to the Preconceptions and Unconscious Bias of HCPs, and Direct and Indirect Financial Toxicity for Patients

HCPs can be subject to unconscious bias, and individual patient characteristics may impact their treatment decisions, resulting in certain patients not receiving the standard of care [54]. Preconceived ideas about the tolerance of older patients to certain mBC treatment regimens may result in their undertreatment. For example, older patients with HER2+ mBC are less likely to receive anti-HER2 therapy and chemotherapy than younger patients [55], while older patients with HR+ HER2− mBC are less likely to be prescribed CDK 4/6 inhibitors [56]. Age-related preconceptions can also impact the care of younger patients with mBC, who may be overtreated with intensive chemotherapy regimens instead of receiving more targeted treatment options [15, 57]. Treatment guidelines recommend that all patients are encouraged to participate in clinical trials [24]. However, in some countries, patients from ethnic minorities are less likely to be informed about clinical trials than Caucasian patients, and those with existing comorbidities often do not meet eligibility criteria [58, 59], thereby limiting access to new treatments for these patients. Comorbidities may also impact treatment decisions in the real-world setting, with patients with comorbidities less likely to receive anti-HER2 therapy for HER2+ mBC than those without coexisting morbid conditions [55]. It is important to consider that some patients may have multiple UPP characteristics that can affect treatment access; for example, many elderly patients with mBC also have comorbidities [60].

Disparities in access to treatment may also be driven by direct and indirect financial toxicity. For example, some patients do not have medical insurance, or are required to pay for their treatment, which can be especially challenging for those with lower incomes [33]. Treatment access may also be hindered by costs associated with transportation to specialist health centres [61], which may be a particular issue for those living in rural locations. Moreover, many patients will reduce their working hours or stop working completely, negatively impacting their ability to pay for certain treatments [33].

##### 3.3.3.1. Existing Initiatives That Aim to Overcome This Need and Solutions Proposed by the UPP Coalition Task Force

Various initiatives have been developed to improve access to diagnostics, effective and safe treatments and high-quality supportive care for patients with mBC [62–64]. The European Commission Initiative on Breast Cancer quality assurance (QA) scheme aims to improve access to quality care in Europe [64]. The scheme defines a set of quality and safety requirements to support HCPs and other stakeholders to provide high-quality breast cancer care, supports the facilitation of the required services and certifies health centres that effectively implement these [64]. The implementation of quality standards can support equitable access to high-quality care for all patients with mBC.

Furthermore, recent efforts have highlighted the economic burden of mBC, as well as ways to support those who are affected by financial toxicity [65]. Financial toxicity associated with mBC was highlighted in an Economist Impact Report published in 2022 [65]. The report investigated and proposed solutions to the challenges faced by patients with mBC and their caregivers in selected upper-middle and high-income countries [65]. It called for flexible workplace policies and highlighted best practices, such as laws in Italy that ensure cancer patients are assigned tasks aligned to their changing capacity without change to their salary [65].

A selection of additional examples of initiatives that aim to improve access to mBC treatment [13] are shown in Table A6. It should be noted that very few initiatives exist to overcome HCP unconscious bias.

The UPP Coalition Task Force suggested several solutions to support equal access to appropriate mBC treatment and care. They agreed that enhancing HCP adherence to mBC guidelines and improving clinical and real-world data collection of treatment outcomes and toxicity data are key to preventing preconceptions and unconscious bias. Furthermore, educating and empowering patients to take a more active role in decision-making may also reduce treatment disparities. Finally, implementation of support services, such as financial support for transportation or treatment costs, or legal counsel for patient discussions with their employer, may help to overcome logistical and financial barriers to treatment for UPPs.

#### 3.3.4. Negative Impact on QoL Resulting From the Limited Uptake of Shared Decision-Making, Low Prioritisation of Patient Preferences and a Lack of Personalised Care

Although patients consider QoL to be one of the most important factors in treatment decision-making [66], physicians often focus solely on efficacy and safety data when choosing treatment regimens, instead of balancing these with the impact of a treatment on patients' QoL [67]. Many patients find it difficult to raise treatment concerns or discuss side effects with their doctor, the impact of which can lower their QoL [68]. This may be particularly challenging for patients with low health education and highlights the importance of HCPs actively asking UPPs about side effects and QoL. A recent survey highlighted that while 89% of oncologists and 92% of nurses feel that they discuss QoL with their patients, a third (34%) of patients with HR+ HER2− mBC reported that their oncologist has never asked about their QoL during follow-up appointments [68].

Furthermore, more patients report QoL to be an important factor in first-line treatment than in later lines of therapy [68]. This emphasises the need for continued discussion about QoL, as patients may deprioritise their QoL in favour of efficacy over time. Furthermore, as the number of available treatment options decreases, patients may believe their options are running out and therefore choose to endure severe side effects.

While international guidelines highlight the importance of shared decision-making in mBC [23], its uptake is low in clinical practice [41, 69]. Implementation is challenging and barriers may be exacerbated for patients who are less likely to engage in conversations with their HCP, such as those with low health knowledge or older patients [31, 39, 41, 70, 71]. This may limit discussion of patients' personal treatment goals during consultations, meaning they are not considered in treatment or end-of-life decision-making. Effective communication, using simple sentences, and clear explanations of the disease, treatment plan and prognosis, could help patients to better understand their situation and meaningfully contribute to conversation with their HCPs [70]. This is particularly important for UPPs; however, many do not receive information in an appropriate format [31]. Finally, the UPP Coalition Task Force highlighted the important role of caregivers in advocating for the treatment preferences of UPPs with mBC, but noted that caregivers may also belong to underserved groups and be facing their own challenges [72].

##### 3.3.4.1. Existing Initiatives That Aim to Overcome This Need and Additional Solutions Proposed and Created by the UPP Coalition Task Force

Various initiatives exist to educate mBC patients and create opportunities for shared decision-making. A selection of examples, some of which are featured in the Hard-to-Reach ABC/mBC Communities Toolkit [13], are listed in Table A7.

In 2023, two innovative resources were co-developed by Pfizer and the Task Force. They are endorsed by the ABC Global Alliance and are available on their website:

• The Visual Treatment Pathway
• The Wellness Chart

###### 3.3.4.1.1. Visual Treatment Pathway

**Figure 5:**
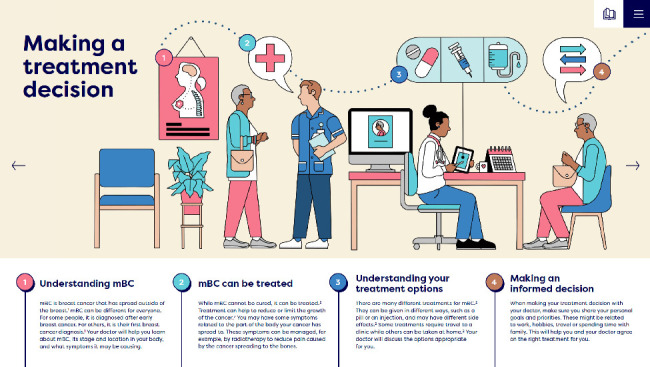
Example page of the Visual Treatment Pathway: making a treatment decision. Reproduction of the Visual Treatment Pathway, with permission from the UPP Coalition Task Force, ABC Global Alliance and Pfizer.

The visual treatment pathway was developed for mBC patients and caregivers with low health literacy and uses simple language and detailed visuals to make the treatment pathway easier to understand. This resource shows information on key stages of mBC treatment, from making decisions to managing side effects, and aims to build patients' confidence in dose reduction as a way to manage toxicity without compromising efficacy (Figure 5).

###### 3.3.4.1.2. Wellness Chart

**Figure 6:**
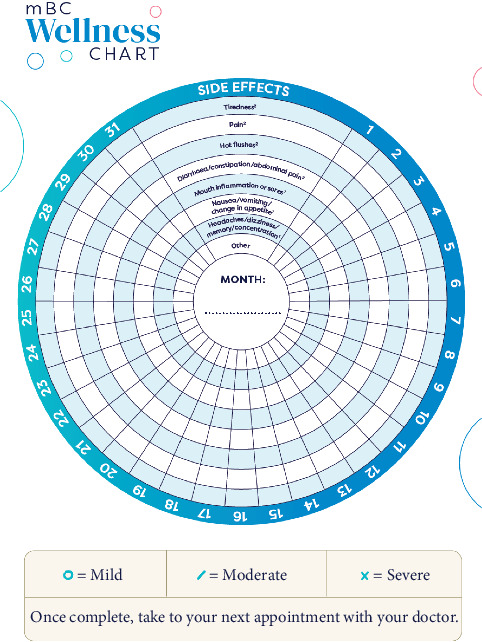
Example page of the Wellness Chart: recording side effects. Reproduction of the Wellness Chart, with permission from the UPP Coalition Task Force, ABC Global Alliance and Pfizer.

The wellness chart was created for patients with low health literacy to track treatment side effects daily (Figure 6). Since many patients do not have the vocabulary to describe the severity or impact of side effects and thereby may suffer unnecessarily, the tool is designed to support patients in consultations with their HCP, who can use it to evaluate their symptoms and determine a management plan.

###### 3.3.4.1.3. Other Proposed Solutions

Experts highlighted that wider implementation of patient navigation services could support UPPs with shared decision-making in mBC. In a recent survey, navigator or social worker follow-up of patients was identified as a strategy to support patients with low health literacy in decisions about their treatment plan [73].

### 3.4. UPPs With mBC in Low- and Middle-Income Countries (LMICs)

Patients with mBC in LMICs experience significantly worse survival outcomes compared with those in high-income countries [2–4]. For UPPs, many of the challenges discussed throughout this article—such as delays in diagnosis, limited availability of specialist support and navigation, and unconscious biases, are exacerbated in LMICs, underscoring the pressing need for solutions in these countries. However, LMIC healthcare system resource and infrastructure may present a challenge for the implementation of existing UPP initiatives, as well as some of the solutions suggested by the UPP Coalition Task Force. For example, almost 90% of the global shortage of nurses reported in 2020 was concentrated in LMICs, limiting the support that can be provided to patients [44, 45]. Consideration must be given to the appropriate adaptation of initiatives to meet the specific needs of UPPs in LMICs and ensure equal provision of quality care for all patients with mBC, regardless of country income level.

## 4. Conclusions

Despite innovation in mBC treatment in the last decade, substantial unmet needs remain for UPPs, who continue to receive unequal access to care. To improve outcomes for these patients, collaboration and action from all stakeholders in the mBC community are needed. The UPP Coalition Task Force identified key areas of focus for improvement and proposed actionable steps to overcome challenges faced by UPPs with mBC globally, including delays in diagnosis, suboptimal use of multidisciplinary care, limited communication between HCPs, unequal access to the most appropriate treatment option and limited uptake of shared decision-making. The UPP Coalition Task Force is committed to continued engagement and collaboration to drive global change in mBC care and to co-create further resources to improve disparities in care for UPPs with mBC.

### 4.1. Call-to-Action

The UPP Coalition Task Force calls on all stakeholders to communicate and collaborate to implement changes, to overcome unmet needs for UPPs with mBC and reduce disparities in care globally.

#### 4.1.1. Educate Primary Care Physicians and Improve Communication Between HCPs

Professional bodies should improve the education of primary care physicians on the signs of mBC and the timeframe of potential disease relapse after early breast cancer, to reduce delays in diagnosis. Healthcare systems should implement better connections between local primary care physicians and specialist cancer centres to share patients' medical information, facilitate timely diagnosis and improve the multidisciplinary care of patients with mBC. HCPs should educate patients with early breast cancer and their caregivers about the possibility of cancer relapse and discuss the importance of attending follow-up appointments. Healthcare teams should follow up with older patients, even after the discontinuation of screening mammograms, and continue to monitor all patients for signs of mBC.

#### 4.1.2. Improve Patient Access to Adequate Treatment Options and Support Services

Healthcare systems, industry organisations and patient advocacy groups should collaborate to disseminate up-to-date and reliable information on mBC treatment options, and clinical trials to the global community. Oncologists should stay abreast of the latest results of clinical trials and real-world studies in mBC and should be aware of the methods and tools available to support consideration of patients' QoL and preferences when making treatment decisions. Patient advocacy groups should educate patients on available treatment options and support them to challenge their HCPs if their treatment plan is not aligned with the latest guidelines or their personal treatment goals. Healthcare systems and policymakers should develop strategies to improve existing support services and increase their availability, including oncology patient navigation, and financial, legal and mental health support. HCPs should be aware of and connect their patients with local patient advocacy groups, to support patients with financial or logistical barriers to accessing optimal treatment.

#### 4.1.3. Enhance Patient and Public Understanding of mBC and its Treatment Options

Healthcare systems, industry organisations and patient advocates should create campaigns to improve public awareness of mBC and reduce stigma associated with the disease, utilising various distribution channels to improve reach. Healthcare systems, industry organisations, politicians, insurance companies, employer associations and patient advocates should collaborate to develop easy-to-understand and culturally competent materials to help patients and caregivers understand mBC, and the resources available for them, and encourage them to express their personal preferences and participate in shared decision-making. HCPs and patient advocacy groups should empower patients to advocate for themselves and gain a deeper understanding of mBC and their treatment plan if they are able and comfortable to do so.

The continued collaboration of all stakeholders is essential to overcome the unmet needs of UPPs with mBC and to drive change in mBC care.

## Figures and Tables

**Table 1:** Number of physicians completing the HR+ HER2− mBC UPP sizing analysis (2020) and quantitative survey in the Ipsos metastatic breast cancer analysis (2022).

Country	Number of physicians completing the survey: HR+ HER2− mBC UPP sizing analysis (2020)	Number of physicians completing the quantitative survey: metastatic breast cancer analysis (2022)
Germany	35	30
France	35	33
Italy	35	36
Spain	35	32
United Kingdom	35	30
Japan	30	28

**Table 2:** UPP characteristics identified during meetings in 2022-2023.

UPP characteristic	Rationale for defining as UPP
Patients with uncontrolled comorbidities	Patients may have limited access to certain treatment options
Patients with limited mobility or access to suitable transportation to a healthcare facility	Patients are at risk of delayed presentation to healthcare appointments after recognition of symptoms or getting lost in follow-up
Patients below 40 years of age	Symptoms may be overlooked by HCPs due to mBC being less common in this age group, therefore delaying diagnosis
Men	Symptoms may be overlooked by HCPs due to breast cancer being uncommon in these patients (about 1% of all cases) [29], therefore delaying diagnosis
Patients who identify as lesbian, gay, bisexual, transgender, queer, intersex or asexual and others (LGBTQIA+)	Patients are at risk of delayed diagnosis due to unconscious bias of HCPs, who may not consider that LGBTQIA+ patients could have mBC
Patients who lack an adequate caregiver or support system	A lack of personal support may mean patients are less likely to detect their symptoms or understand their treatment plan
Single or divorced patients	A lack of financial support may mean patients are unable to attend medical appointments or pay for treatments and associated expenses
Patients who mistrust conventional treatments	Patients may not receive timely diagnosis or optimal treatment, as they often try alternative treatment options before seeking help from primary care physicians or oncologists
Survivors of early breast cancer	These patients are at risk of delayed diagnosis, as they may consider themselves ‘cured' and not be aware that breast cancer can reoccur or may delay attending healthcare appointments due to fear mBC
Mental health patients	Patients may be less likely to recognise their symptoms, resulting in delayed diagnosis
Hyperinformed patients (those who combine the opinions of several HCPs and their own research to select their preferred treatment regimen)	These patients may or may not take the treatment as prescribed and may instead follow their own judgement (sometimes taking supplements not prescribed by their physicians) which may put them at risk of not receiving the optimal treatment option
Patients without health insurance	Patients may not be able to pay for certain treatment options

Abbreviations: HCP = healthcare professional, mBC = metastatic breast cancer and UPP = underserved patient population.

**Table 3:** Members of the UPP Coalition Task Force meetings from 2022 to 2023.

Stakeholder type	Europe/Japan task force (2 meetings)	US task force (1 meeting)	Global task force (1 meeting)
Oncologist	9	7	8
Patient advocacy group representative/patient advocate	2	3	3
ABC Global Alliance President	1		1
European Cancer Organisation: Policy expert			2
Oncology navigator			1
Nurse		1	1
Social worker			1

**Table 4:** Proportion of HCPs who believe different factors impact access to breast cancer diagnosis in their countries [14].

	Germany (%)	France (%)	Italy (%)	Spain (%)	UK (%)	Japan (%)
Fear of the outcome or denial that there may be a problem	77	48	58	50	70	68
Lower health knowledge	77	58	39	53	63	68
Age 75+ years	70	48	39	69	70	57
Underestimation of the severity of the issue	53	64	44	47	50	54
Living in a rural area	50	52	50	50	43	32
Lower income level	63	55	33	25	43	46
Ethnic, religious, indigenous or native populations and/or other minorities	67	48	39	28	47	18
Social stigma	43	36	22	16	40	39
Marital status (divorced or widowed women)	33	33	11	16	30	29

*Note:* Findings of the Metastatic Breast Cancer—Barriers to Early Breast Cancer Detection Analysis conducted by Ipsos in 2022.

Abbreviation: UK = United Kingdom.

**Table 5:** Proportion of HCPs who believe different patient characteristics impact their decision to prescribe CDK 4/6 inhibitors to patients with HR+ HER2− mBC [14].

	Germany (%)	France (%)	Italy (%)	Spain (%)	UK (%)	Japan (%)
Comorbidities/pre-existing conditions	50	64	47	50	70	57
No caregiver/support network	50	45	44	41	47	25
Patient age (75+ years)	27	45	36	38	53	43
Income level	37	36	19	22	33	64
Health knowledge/language barriers of the patient (ability to understand the treatment)	50	45	22	38	17	36

*Note:* Findings of the Metastatic Breast Cancer—Barriers to Early Breast Cancer Detection Analysis conducted by Ipsos in 2022.

Abbreviation: UK = United Kingdom.

**Table 6:** Examples of initiatives developed to improve public and HCP awareness of mBC.

Name of initiative	Organisation	Country	Aim
I Am Advanced Breast Cancer [74]	ABC Global Alliance	Global	Raise awareness of mBC and its associated challenges through real patient stories
ABC Project^†^ [13]	Cancer Solutions KK	Japan	Educate patients with mBC, caregivers and HCPs about mBC treatment, and psychological, social and financial issues through online seminars and learning modules
Mamma Mia!—The Breast Cancer Magazine [75]	Mamma Mia! German Breast Cancer Association	Germany	Improve awareness of breast cancer in Germany and to provide information about the disease, treatments and scientific advances in an easily understandable format for patients with breast cancer
Metastatic Breast Cancer Awareness Day [76]	Metastatic Breast Cancer Network	US	Increase recognition and awareness of patients with mBC
Women Don't Die From Breast Cancer [77]	METAvivor	US	Raise awareness of mBC in order to increase research funding
I Am The 31 campaign [78]	MetUpUK	UK	Raise awareness of mBC by highlighting real stories of patients living with mBC
SBC Infographics^†^ [79] (available in 13 different languages)	MetUpUK	Global	Improve awareness of mBC signs and symptoms in the public and increase patient presentation to the healthcare system
The Invisible Woman 2.0 2020 Report [80]	Novartis	Europe	Raise awareness of the impact of mBC to patients and society, and highlight the key challenges people living with mBC face
Spanish-language BC Patient Education Novelas^†^ [13]	Pfizer, SHARE/LATINASHARE	Global	Raise awareness and educate people about mBC through depicting the journey of a woman receiving mBC diagnosis
Care-A-Van Breast Cancer Project^†^ [12]	Pfizer Malaysia	Malaysia	Improve early diagnosis in urban low socioeconomic communities, to provide information about mBC and to support patients throughout their treatment
Talking to your kids about breast cancer—a guide for mothers^†^ [12]	Rethink Breast Cancer	Canada	Support young mothers with educating their children on mBC and talking about treatment and end of life

*Note:* The table contains selected examples of global and local initiatives developed to improve mBC awareness and represents a nonexhaustive list. The initiatives that are featured in the ABC Global Alliance and Pfizer's Hard-to-Reach ABC/mBC Communities toolkit are denoted with a † symbol.

Abbreviations: HCP = healthcare professional, mBC = metastatic breast cancer, UK = United Kingdom and US = United States.

**Table 7:** Examples of resources and initiatives to improve patient navigation services.

Name of resource/initiative	Organisation	Country	Aim
Building Expertise, Advocacy, and Capacity for Oncology Navigation (BEACON) [81]	American Cancer Society	Global	Address health disparities in patient cancer navigation programmes in low- and middle-income countries and other relevant settings globally, and to support and promote the integration of patient navigation into cancer care
Metastatic Breast Cancer Pathways Resource Guide for Navigators [82]	Pfizer	US	Support the work of mBC patient navigators by providing information on mBC, treatment and care, and links to relevant resources for the navigators, patients and caregivers
The Foundations of Cancer Care^†^ [13]	The Institute of Cancer Research, Ocean Road Cancer Institute, The Royal Marsden, Breast Care International, Macmillan Cancer Support	Ghana and Tanzania	Develop a culturally-sensitive training programme for nurses and peer supporters involved in the delivery of cancer care
Patient Navigation Program^†^ [13]	University of Nigeria Teaching Hospital Cancer Support Group	Nigeria	Provide emotional and psychosocial support, and education for patients by newly established patient navigation services

*Note:* The table contains selected examples of global and local initiatives developed to improve patient navigation services in mBC care and represents a nonexhaustive list. The initiatives that are featured in the ABC Global Alliance and Pfizer's Hard-to-Reach ABC/mBC Communities toolkit are denoted with a † symbol.

Abbreviation: mBC = metastatic breast cancer.

**Table 8:** Initiatives that aim to improve the quality of care for patients with mBC.

Name of report/initiative	Organisation	Country	Aim
The Truth About Working With ABC [83]	ABC Global Alliance, Working With Cancer	Global	Improve awareness of the challenges associated with employment for people with mBC and support patients and their employers to navigate working with mBC
Humanitarian Partnership for Access to Cancer Treatment (PACT) [62]	ABC Global Alliance, The Max Foundation, American Society for Clinical Pathology, Cepheid, Novartis AG	Global (low- and middle-income countries)	Enhance treatment accessibility in low- and middle-income countries for patients with HR+ HER2− mBC
National Cancer Plan [63]	Bundesministerium für Gesundheit	Germany	Improve early cancer detection and diagnosis, cancer care structures, access to treatments and supportive care
Male Breast Cancer Global Alliance^†^ [12]	Male Breast Cancer Global Alliance	US, Australia, South Africa, UK, Kenya	Improve access to research, clinical trials and appropriate treatment options for men with mBC, and to overcome increasing mortality rates in this group of patients

*Note:* The table contains selected examples of global and local initiatives developed to improve the quality of mBC care and represents a nonexhaustive list. The initiatives that are featured in the ABC Global Alliance and Pfizer's Hard-to-Reach ABC/mBC Communities toolkit are denoted with a † symbol.

Abbreviations: EU = European Union, mBC = metastatic breast cancer, UK = United Kingdom and US = United States.

**Table 9:** Examples of initiatives that aim to support shared decision-making and improve the QoL of patients with mBC.

Name of initiative	Organisation/creator	Country	Aim
The Dandelion Toolkit^†^ [84]	Dr Ellsworth-Beaumont, Metastatic Breast Cancer Alliance, ABC Global Alliance, Pfizer	Global	Provide a simple visual aid for patients to help them understand mBC and the treatment options available to them. The toolkit also includes tools to assess and discuss the QoL of patients
Let the Hope Blossom^†^ [12]	Europa Donna Turkey, Turkish Medical Oncology Association, Pfizer Turkey	Turkey	Improve the QoL of patients by providing psychological support and by raising awareness about the importance of the QoL of patients with mBC
You Have a Choice Campaign^†^ [12]	Federation of Associations “Amazons, Spa for Cancer,” Pfizer	Poland	Educate patients about the benefits of CDK 4/6 inhibitors and to raise awareness about the importance of involving patients in treatment decisions
Consultation in pictures^†^ [12]	Médipôle Hôpital Mutualiste de Lyon-Villeurbanne	France	Help HCPs explain the diagnosis, treatment options, steps of the treatment pathway and support available to patients who do not speak French well in a visual format
Vision zero initiative [85]	Nationale Dekade Gegen Krebs Unterstützer, Aktionsbündnis Patientensicherheit	Germany	Reduce the number of avoidable cancer deaths to zero through increasing research, improving cancer care and increasing patient involvement in research and their treatment
Young Cancer Portal [86]	German Foundation for Young Adults with Cancer	Germany	Improve shared decision-making in the community on an individual basis
Moments that Count campaign [87]	Novartis	UK	Provide information and helpful resources for patients with breast cancer, including resources that aim to support patients in their discussions with HCPs and encourage engagement in shared decision-making
MBC Dictionary^†^ [13]	Pfizer, Breastcancer.org	US	Improve patient understanding and improve patient engagement in conversations with their HCPs by providing context to and explanations of key terms used during mBC consultations
Reframing advanced breast cancer^†^ [12]	Venezuelan Breast Cancer Research and Education Foundation	Venezuela	Educate patients about mBC and treatment options for the disease, and to empower them to engage in shared decision-making through a social media campaign and web-based educational tool

*Note:* The table contains selected examples of global and local initiatives developed to improve the uptake of shared decision-making and the QoL of patients with mBC and represents a nonexhaustive list. The initiatives that are featured in the ABC Global Alliance and Pfizer's Hard-to-Reach ABC/mBC Communities toolkit are denoted with a † symbol.

Abbreviations: HCP = healthcare professional, mBC = metastatic breast cancer, QoL = quality of life, UK = United Kingdom and US = United States.

## Data Availability

The data that support the findings of this study are not available to the public.

## Nomenclature

ABC: Advanced breast cancer
CDK: Cyclin-dependent kinase
HCP: Healthcare professional
mBC: Metastatic breast cancer
MDT: Multidisciplinary team
PARP: Polyadenosine diphosphate ribose polymerase
PI3K: Phosphatidylinositol 3-kinase
QoL: Quality of life
UPP: Underserved patient population
